# Lytic Cell Death Mechanisms in Human Respiratory Syncytial Virus-Infected Macrophages: Roles of Pyroptosis and Necroptosis

**DOI:** 10.3390/v12090932

**Published:** 2020-08-25

**Authors:** Lori Bedient, Swechha Mainali Pokharel, Kim R. Chiok, Indira Mohanty, Sierra S. Beach, Tanya A. Miura, Santanu Bose

**Affiliations:** 1 Department of Veterinary Microbiology and Pathology, College of Veterinary Medicine, Washington State University, Pullman, WA 99164, USA; lori.bedient@wsu.edu (L.B.); s.mainalipokharel@wsu.edu (S.M.P.); k.chiokcasimiro@wsu.edu (K.R.C.); indira.mohanty@wsu.edu (I.M.); 2 Department of Biological Sciences, University of Idaho, Moscow, ID 83844, USA; beac7858@vandals.uidaho.edu (S.S.B.); tmiura@uidaho.edu (T.A.M.)

**Keywords:** RSV, respiratory syncytial virus, macrophages, necroptosis, pyroptosis, lytic cell death

## Abstract

Human respiratory syncytial virus (RSV) is the most common cause of viral bronchiolitis and pneumonia in infants and children worldwide. Inflammation induced by RSV infection is responsible for its hallmark manifestation of bronchiolitis and pneumonia. The cellular debris created through lytic cell death of infected cells is a potent initiator of this inflammation. Macrophages are known to play a pivotal role in the early innate immune and inflammatory response to viral pathogens. However, the lytic cell death mechanisms associated with RSV infection in macrophages remains unknown. Two distinct mechanisms involved in lytic cell death are pyroptosis and necroptosis. Our studies revealed that RSV induces lytic cell death in macrophages via both of these mechanisms, specifically through the ASC (Apoptosis-associated speck like protein containing a caspase recruitment domain)-NLRP3 (nucleotide-binding domain, leucine-rich-containing family, pyrin domain-containing-3) inflammasome activation of both caspase-1 dependent pyroptosis and receptor-interacting serine/threonine-protein kinase 3 (RIPK3), as well as a mixed lineage kinase domain like pseudokinase (MLKL)-dependent necroptosis. In addition, we demonstrated an important role of reactive oxygen species (ROS) during lytic cell death of RSV-infected macrophages.

## 1. Introduction

Respiratory syncytial viruses (RSV) are among the most common causes of viral pneumonia in neonatal, elderly, and immunocompromised humans worldwide. Human RSV infection in children under five years of age leads to over three million hospitalizations and nearly 60,000 in-hospital deaths annually [1]. Elderly or immunocompromised individuals such as organ transplant recipients, HIV (Human Immunodeficiency virus) -infected persons, or those with comorbidities such as asthma or chronic obstructive pulmonary disease are considered especially prone to infection and are more likely to be hospitalized [2,3]. Antiviral treatment for RSV infection is largely limited by expense and lack of efficacy data in those populations most at risk. Despite extensive research, an effective vaccine remains elusive [4,5].

RSV-induced pneumonia results from an exaggerated pro-inflammatory response triggered by excessive cytokine and chemokine release from infected inflammatory immune cells [6,7,8,9,10]. RSV-infected alveolar and exudative macrophages within the airway are instrumental in the induction of these excessive pro-inflammatory cytokines leading to fulminant pneumonia. In addition to cytokine and chemokine release, lytic cell death of infected cells including macrophages has similarly been shown to amplify the inflammatory response following infection with various pathogens [11,12,13,14]. In the case of RSV, cell debris generated by this cell death results in the physical obstruction of small airways by accumulating cellular fragments and leads to the classic lesion of bronchiolitis [15]. These cellular fragments then act as potent danger associated molecular patterns (DAMPs) to further amplify the “cytokine storm” and existing airway inflammation [8,16,17].

Lytic cell death is an inflammatory cell death caused primarily by two cellular mechanisms: pyroptosis and necroptosis [12,13,18]. Pyroptosis occurs as a sequela of inflammasome activation through the downstream activity of Caspase-1 on Gasdermin D, the central component of pyroptotic membrane pore formation leading to osmotic-induced membrane rupture [12,19]. Caspase-1 also causes cleavage of pro-IL-1β into mature IL-1β, a process not intrinsically linked to potential pyroptotic cell death. Necroptosis occurs through the activity of the necrosome, a complex of three key proteins: receptor-interacting serine/threonine-protein kinase 1 (RIPK1), receptor-interacting serine/threonine-protein kinase 3 (RIPK3), and mixed lineage kinase domain like pseudokinase (MLKL) [13,20,21]. However, necroptosis can also occur via a RIPK1-independent mechanism following activation of the RIPK3-MLKL pathway [22,23,24]. Activation of MLKL occurs via phosphorylation by RIPK3, leading to migration of the activated form of MLKL to the plasma membrane to create pores for osmotic rupture.

Our lab has previously demonstrated the role of RSV infection in inflammasome activation and IL-1β release in macrophages [25,26]. Since inflammasome activation serves as a precursor for pyroptosis-mediated lytic cell death [12], these results suggest that pyroptosis may be involved in lytic cell death of RSV-infected macrophages. Although recent studies have demonstrated necroptosis as the functional pathway in recruited neutrophilic cell death during RSV infection [27], lytic cell death mechanisms are both pathogen and cell-specific. While macrophages play a pivotal role in the development of airway inflammation during RSV infection [28,29], the specific roles of necroptosis and pyroptosis in inducing lytic cell death in RSV-infected macrophages are not presently known. Additionally, reactive oxygen species (ROS) play an important role in RSV infection and pathogenesis [30,31]. Our lab and others have demonstrated a role of ROS in regulating infection and innate immune responses in RSV-infected macrophages [25,32]. However, the role of ROS in lytic cell death during RSV infection has not yet been investigated.

In the present study, we demonstrated that RSV-infected macrophages induce both pyroptosis and necroptosis. Pyroptosis was induced via Caspase-1 following activation of the ASC-NLRP3 inflammasome. We also demonstrated that necroptosis induces lytic cell death in RSV-infected macrophages through the RIPK3-MLKL pathways. Moreover, we show that ROS play a key role in positively regulating lytic cell death of RSV-infected macrophages. These results indicate that both pyroptosis and necroptosis are involved during ROS-dependent RSV-induced lytic cell death in macrophages.

## 2. Materials and Methods

### 2.1. Virus and Cells

Human respiratory syncytial virus (RSV; A2 strain) was purified, as described previously [25,26,33,34,35]. Recombinant human RSV expressing mKate2 protein (RSV-mKate2) was propagated from pSynk-A2 as described previously [36,37]. pSynk-A2 and helper plasmids were provided by Dr. Martin Moore (Emory University) and BSRT7/5 cells were provided by Dr. Ursula Buchholz (National Institutes of Health). The human monocyte cell line (THP-1) (ATCC, Manassas, VA, USA; catalog no. TIB-202) was cultured in 1640 RPMI, 10% FBS (Fetal Bovine Serum), 100 IU/mL Penicillin, 100 µg/mL Streptomycin, 1 mM sodium pyruvate, 10 mM HEPES [4-(2-hydroxyethyl)-1-piperazineethanesulfonic acid], and 50 µM β-mercaptoethanol (Sigma Aldrich, St. Louis, MO, USA). THP-1 cells were plated in 48 well plates at a concentration of 2 × 10^5^ cells/well, differentiated by treatment with 100 nM phorbol 12-myristate 13-acetate (PMA) (Sigma Aldrich), and allowed to incubate for 24 h. Undifferentiated, unattached cells were then removed by washing with PBS after 24 h and cells were allowed to incubate for an additional 24 h in the PMA-free medium prior to infection. ASC-deficient THP-1 (THP-1-ASC-def) cells (catalog # thp-dasc), NLRP3-deficient THP-1 (THP-1-NLRP3-def) cells (catalog # thp-dnlp), and positive-control THP-1 wild-type (THP-1-WT) cells (catalog # thp-null) were purchased from InvivoGen, San Diego, CA, USA.

### 2.2. Cell Treatment and Infection

Differentiated THP-1 cells were pre-treated with the reconstitution vehicle (vehicle control) or a specific inhibitor for 2 h. After 2 h pre-treatment, cells were infected with RSV (Multiplicity of infection or MOI = 1) as described previously [25,26,33,34,35]. Briefly, cells were incubated with RSV for 1.5 h in a serum-free, antibiotic-free OPTI-MEM media (GIBCO, Waltham, MA, USA). Following adsorption, cells were washed with PBS and infection was continued in the presence of serum containing complete media with either vehicle control or the inhibitors. In order to analyze IL-1β production following RSV infection, THP-1-WT, THP-1-ASC-def, and THP-1-NLRP3-def cells were infected with RSV (MOI = 1) for 16 h. Cells were treated with the ROS inhibitor diphenyleneiodonium chloride (Enzo, Farmingdale, NY, USA, catalog # BML-CN240-0010) (10 µm), Caspase-1 inhibitor Ac-YVAD-CHO (Enzo, catalog # ALX-260-027) (20 or 40 µm), Z-VAD-FMK Pan-caspase inhibitor (Invivogen, catalog # tlrl-vad) (25 or 50 µm), RIPK3 inhibitor GSK ‘872 (Tocris, Bristol, UK, catalog #6492) (20 or 40 µM), MLKL inhibitor necrosulfonamide (Tocris, catalog # 5025) (10 or 20 µm). Caspase-3 inhibitor Ac-DEVD-CHO (Sigma Aldrich, catalog # A0835) (100 µM). IL-1β production from THP-1-WT, THP-1-ASC-def, and THP-1-NLRP3-def cells was measured following treatment of cells with LPS (Invivogen, catalog # tlrl-eblps) (100 ng/mL) for 4 h, followed by nigericin (Invivogen, catalog # tlrl-nig) (15 µM) treatment for 30 min. In some experiments DMSO (Dimethyl sulfoxide) (Sigma Aldrich) was used as a vehicle and vehicle control.

### 2.3. Western Blotting

Cell lysates collected from mock and RSV-infected macrophages were subjected to Western Blot analysis to detect caspase-3 cleavage following caspase-3 activation. Caspase-3 cleavage was analyzed by performing Western blotting with caspase-3 antibody (cell signaling technology, Danvers, MA, USA, catalog # 9662). The actin antibody was purchased from Bethyl Laboratories, Montgomery, TX, USA (catalog # A300–485A). In some experiments, THP-1 cells were pre-treated with caspase-3 inhibitor Ac-DEVD-CHO (100 µM) for 2 h, followed by 48 h treatment with 1 µM, 2 µM, and 5 µM Thapsigargin (Cayman chemicals, Ann Arbor, MI, USA, catalog # 10522). THP-1 cells infected with RSV-mKate2 (MOI = 2) were subjected to Western blot analysis with anti-RFP antibody (RF5R) (ThermoFisher Scientific, Waltham, MA, USA, catalog # MA5-15257).

### 2.4. ELISA

IL-1β levels in the medium supernatant were assessed by using an IL-1β specific ELISA kit (Invitrogen, Carlsbad, CA, USA, catalog # 88-7261-22).

### 2.5. LDH Assay

The LDH-Cytotoxicity Assay Kit II was purchased from Biovision, Milpitas, CA, USA (Catalog # K313-500) and used per manufacturer instructions. Based on the manufacturer’s instruction, we used cell lysates as a high control. For the assay, medium supernatant collected from the mock infected and RSV-infected cells were incubated with LDH Reaction Mix. At the high control (i.e., the cell lysates as pointed above) absorbance of 2.0 OD (optical density), the absorbance of the experimental samples (i.e., the medium supernatants from mock and RSV-infected cells) was measured at 450 nm by using a micro-plate reader. LDH release values shown in Figures 2b, 3a,b, 4a,f and 5 were calculated based on the following formula:

(a) “RSV + vehicle” values were calculated by subtracting OD of vehicle treated RSV-infected cells from OD of vehicle treated mock cells.
(b) “RSV + inhibitor” values were calculated by subtracting OD of inhibitor treated RSV-infected cells from OD of inhibitor treated mock cells.

The LDH release value shown in Figure 4d was calculated based on the following formula:

(a) “Null + RSV” value was calculated by subtracting OD of RSV-infected null cells from OD of mock null cells.
(b) “ASC def + RSV” value was calculated by subtracting OD of RSV-infected ASC def cells from OD of mock ASC def cells.
(c) “NLRP3 def + RSV” value was calculated by subtracting OD of RSV-infected NLRP3 def cells from OD of mock NLRP3 def cells.

### 2.6. Statistical Analysis

LDH release data were statistically analyzed using the Student’s *t*-test in Graphpad PRISM software (version 8.3.0).

## 3. Results

### 3.1. RSV Induces Lytic Cell Death in Macrophages

**Figure 1:**
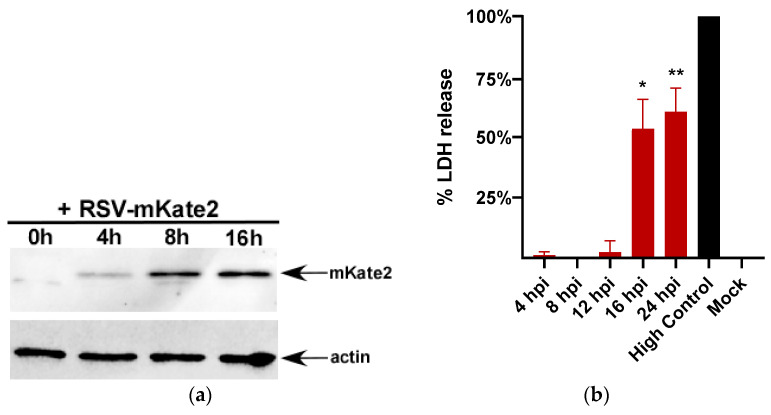
Lactate dehydrogenase (LDH) release during respiratory syncytial virus (RSV) infection of the human monocyte cell line (THP-1) macrophages. (**a**) THP-1 macrophages infected with RSV-mKate2 (MOI = 2) for 0 h–16 h were subjected to Western blotting with anti-RFP antibody. Western blot data shown is representative of three independent experiments with similar results. (**b**) THP-1 macrophages were infected with RSV (MOI = 1) and LDH release was measured at 4 h, 8 h, 12 h, 16 h, and 24 h post infection (*n* = 16 technical replicates from two independent experiments). % LDH release was calculated by using high control (cell lysate) value as 100% LDH release. * *p* and ** *p* ≤ 0.05 compared to mock using a Student’s *t*-test.

RSV mediated lytic cell death has not been investigated in macrophages. Therefore, we used the human monocyte THP-1 macrophage cell line to study lytic cell death following RSV infection. First, we evaluated THP-1 cell susceptibility to RSV infection by infecting these cells with a recombinant RSV that expresses mKate2 protein (RSV-mKate2) [36,37]. RSV infection was monitored in THP-1 cells by performing Western blotting with anti-RFP antibody which detects the mKate2 protein. THP-1 cells are susceptible to RSV infection since we detected expression of mKate2 in THP-1 cells as early as 4 h post-infection (Figure 1a).

Lactate dehydrogenase (LDH) is released from cells as a consequence of lytic cell death and can be readily quantified using an LDH assay. Therefore, lytic cell death was measured by analyzing the levels of LDH in the medium supernatants of RSV-infected THP-1 macrophages. In order to study lytic cell death kinetics following RSV infection, we measured LDH release from RSV-infected THP-1 cells at 4 h, 8 h, 12 h, 16 h, and 24 h post infection. Our results revealed 16 h post-infection as an optimal time point for LDH release from RSV-infected cells (Figure 1b) and we therefore chose a 16h post-infection time point for subsequent experiments.

### 3.2. RSV Mediated LDH Release Is Mainly Due to Lytic Cell Death

**Figure 2:**
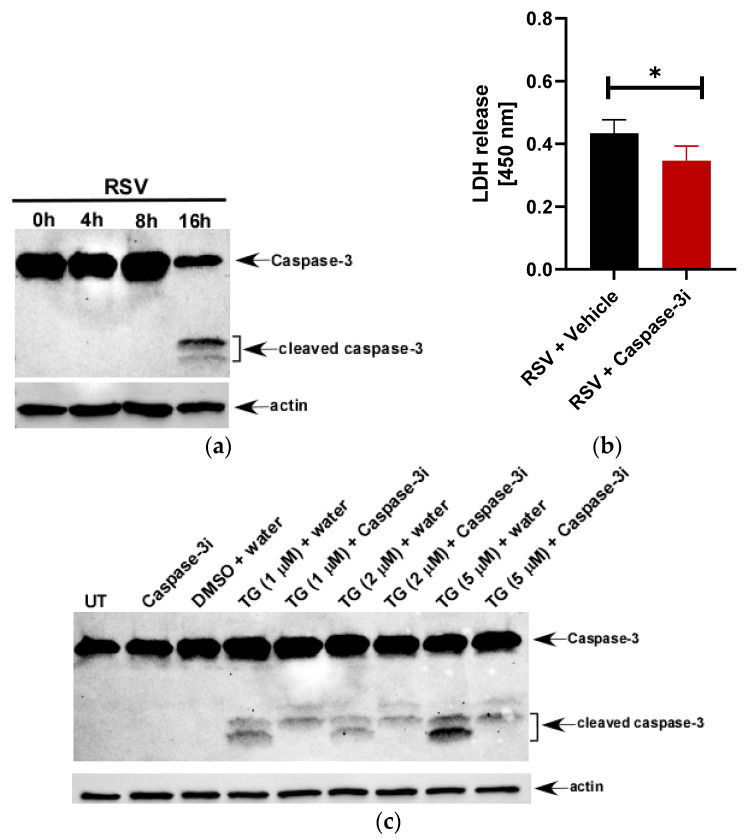
LDH release during RSV infection of macrophages is primarily due to lytic cell death. (**a**) Human THP-1 macrophages infected with RSV (MOI = 1) for 0 h–16 h were subjected to Western blotting with anti-caspase-3 antibody that can detect both the full length and cleaved fragments of activated caspase-3. (**b**) Human THP-1 macrophages were infected with RSV (MOI = 1) in the presence of either vehicle (water) or the caspase-3 inhibitor Ac-DEVD-CHO (100 µM). LDH release was measured (at OD of 450 nm) at 16 h post-infection infection (*n* = 16 technical replicates from two independent experiments). * *p* ≤ 0.05 using a Student’s *t*-test. (**c**) Untreated (UT) and THP-1 cells treated with indicated concentrations of thapsigargin (TG) in the presence of either water (vehicle control) or caspase-3 inhibitor Ac-DEVD-CHO (100 µM) for 48 h were subjected to Western blotting with anti-caspase-3 antibody that can detect both the full length and cleaved fragments of activated caspase-3. TG is soluble in DMSO and therefore, a lane for DMSO + water was included for the Western blot analysis. Western blot data is representative of two-three independent experiments with similar results. Caspase-3i: caspase-3 inhibitor Ac-DEVD-CHO.

Cell death may occur from lytic (pyroptosis and necroptosis) or non-lytic (apoptosis) mechanisms. Since apoptosis is a non-lytic mechanism of cell death, LDH release from the cytoplasm is considered minimal. Although the apoptosis inhibitor Ac-DEVD-CHO efficiently blocks apoptosis in macrophage cell lines like THP-1 [38], it has no effect on LDH-releasing macrophages undergoing lytic cell death [39,40]. This key distinction allows for the in vitro use of an LDH assay to broadly characterize non-lytic from lytic cell death mechanisms. Nevertheless, the role of apoptosis in LDH release was explored in RSV-infected THP-1 macrophages through inhibition of caspase-3, the key executioner apoptosis caspase in both extrinsic and intrinsic pathways. Caspase-3 is indeed activated by RSV in THP-1 cells as we observed caspase-3 cleavage, a hallmark of caspase-3 activation, in RSV-infected cells (Figure 2a). Inhibition of caspase-3 by Ac-DEVD-CHO led to only 18% reduction in LDH release from RSV-infected THP-1 cells (Figure 2b). The activity of Ac-DEVD-CHO was confirmed through Western blot analysis showing marked inhibition of caspase-3 cleavage in the presence of the apoptosis inducer thapsigargin (TG) (Figure 2c).

The loss of LDH release following caspase-3 inhibition during RSV infection could be attributed to possible mechanistic crosstalk between apoptosis and necroptosis and/or induction of secondary necroptosis. In the absence of scavenger cells to complete phagocytosis of apoptosis-initiated cells, cells may undergo secondary necroptosis with similar morphological and chemical changes as found in primary necroptosis: cell membrane permeability, lysosomal rupture, and cellular swelling [41,42,43,44]. Caspase-8 activation can induce caspase-3 dependent apoptosis but can also induce caspase-3 cleavage of Gasdermin E similar to that seen in caspase-1 dependent pyroptosis through the cleavage of Gasdermin D [45]. This form of pyroptosis, termed incomplete pyroptosis, may account for the loss of LDH release when caspase-3 is inhibited. Nevertheless, our studies demonstrated that LDH release from RSV-infected macrophages is primarily due to lytic forms of cell death (i.e., pyroptosis and necroptosis) rather than apoptosis.

### 3.3. Roles of RIPK3-MLKL in RSV-Induced Necroptosis

**Figure 3:**
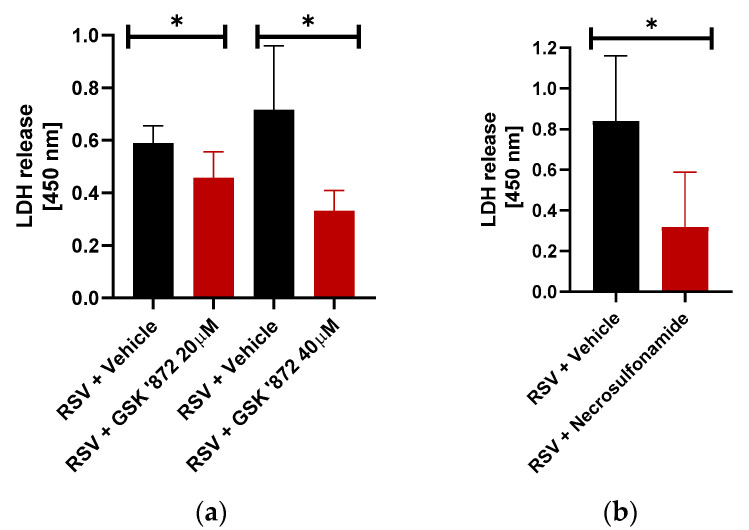
Necroptotic cell death of RSV-infected macrophages via RIPK3-MLKL pathway. (**a**) Human THP-1 macrophages were infected with RSV (MOI = 1) in the presence of either vehicle (DMSO) or RIPK3 inhibitor GSK’872 (20 µM and 40 µM). LDH release was measured (at OD of 450 nm) at 16 h post-infection (*n* = 16 technical replicates from two independent experiments). * *p* ≤ 0.05 using a Student’s t-test. (**b**) Human THP-1 macrophages were infected with RSV (MOI = 1) in the presence of either vehicle (DMSO) or MLKL inhibitor Necrosulfonamide (20 µM). LDH release was measured (at OD of 450 nm) at 16h post-infection (*n* = 14 technical replicates from two independent experiments). * *p* ≤ 0.05 using a Student’s *t*-test.

First, we investigated the potential role of necroptosis during lytic cell death of RSV-infected macrophages. Necroptosis occurs through the activity of the receptor-interacting serine/threonine-protein kinase 3 (RIPK3) and mixed lineage kinase domain like pseudokinase (MLKL) pathway [13,46]. Activation of RIPK3 recruits and phosphorylates MLKL [47], which oligomerizes to form a pore-forming structure that migrates to the cell membrane and initiates cell death. We therefore used RIPK3 and MLKL inhibitors individually to identify the key mechanistic components of the necroptotic pathway. To investigate RIPK3′s role, we treated THP-1 macrophages with the RIPK3 specific inhibitor GSK’872 which binds directly to its kinase domain. GSK’872 treatment resulted in a dose-dependent, significant 17% and 54% reduction in lytic cell death at the 20 µM and 40 µM concentrations, respectively (Figure 3a). Conversely, inhibition of RIPK1 by necrostatin-1 marginally reduced LDH release from RSV-infected macrophages (data not shown). This result suggests RIPK3 is the major kinase during RSV-mediated necroptosis induction in macrophages.

Next, the effect of the MLKL inhibition was investigated through the treatment of THP-1 cells with the MLKL inhibitor necrosulfonamide. While necrosulfonamide does not prevent phosphorylation of MLKL by RIPK3, it does bind to MLKL at its cysteine residue site and prevents migration of the polymerized MLKL complex to the cell membrane, thereby inhibiting cell lysis. Treatment of the cells with necrosulfonamide resulted in a 64% reduction in lytic cell death (Figure 3b). Thus, our results with necrosulfonamide demonstrated a positive regulatory role of MLKL during lytic cell death of RSV-infected macrophages.

### 3.4. RSV Induces Caspase-1 Dependent Pyroptosis

**Figure 4:**
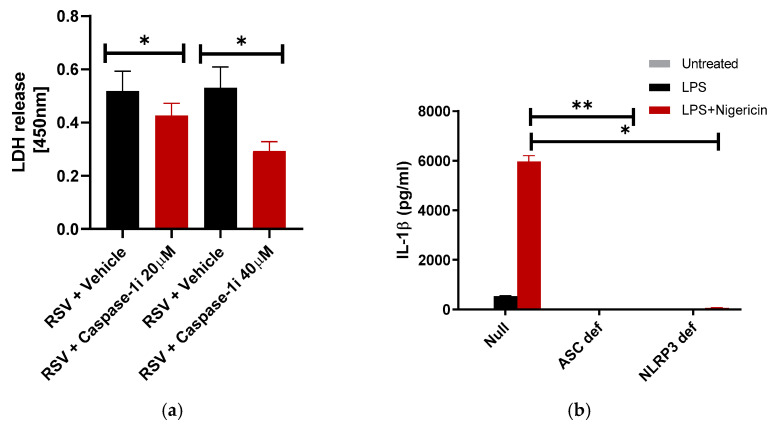

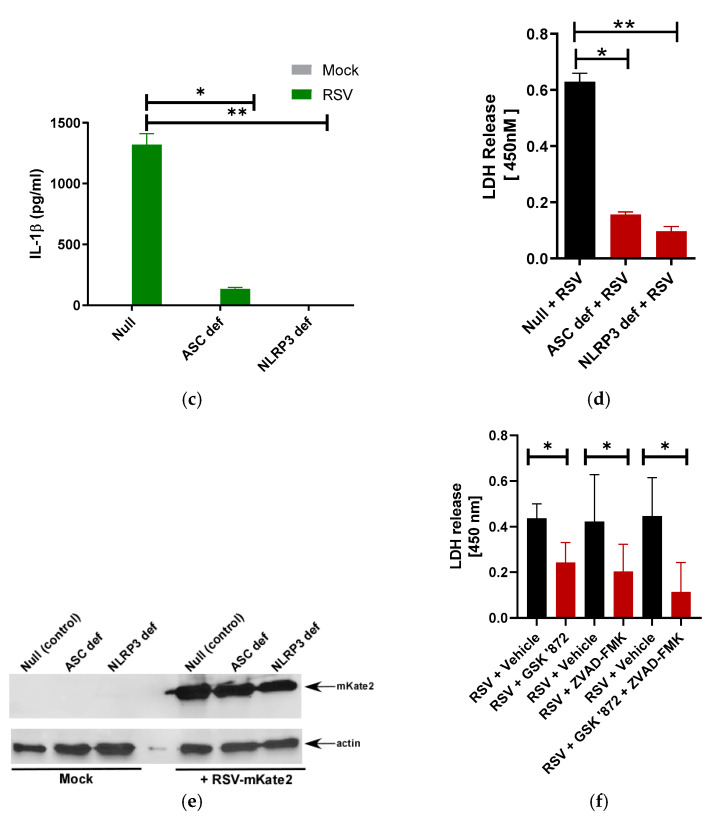
Caspase-1 and ASC-NLRP3 inflammasome is required for pyroptotic cell death of macrophages during RSV infection. (**a**) Human THP-1 macrophages were infected with RSV (MOI = 1) in the presence of either vehicle (DMSO) or caspase-1 inhibitor (caspase-1i) Ac-YVAD-CHO (20 µM and 40 µM). LDH release was measured (at OD of 450 nm) at 16 h post-infection infection (*n* = 12 technical replicates from two independent experiments). * *p* ≤ 0.05 using a Student’s *t*-test. (**b**) Wild-type (null), ASC deficient (ASC def), and NLRP3 deficient (NLRP3 def) THP-1 macrophages were treated with LPS (100 ng/mL) for 4 h, followed by 30 min treatment with nigericin (15 µM). IL-1β release was measured by ELISA (*n* = 16 technical replicates from two independent experiments). * *p* and ** *p* ≤ 0.05 using a Student’s *t*-test. (**c**) Null, ASC def, and NLRP3 def THP-1 macrophages were infected with RSV (MOI = 1). At 16 h post-infection, IL-1β release was measured by ELISA (*n* = 16 technical replicates from two independent experiments). * *p* and ** *p* ≤ 0.05 using a Student’s *t*-test. (**d**) Null, ASC def, and NLRP3 def THP-1 macrophages were infected with RSV (MOI = 1). LDH release was measured (at OD of 450 nm) at 16 h post-infection infection (n = 16 technical replicates from two independent experiments). * *p* and ** *p* ≤ 0.05 using a Student’s *t*-test. (**e**) Null, ASC def, and NLRP3 def THP-1 macrophages were infected with RSV-mKate2 (MOI = 2) for 16 h. Equal levels of total protein from the cell lysates of Null, ASC def, and NLRP3 def cells were subjected to Western blotting with anti-RFP antibody. Western blot data shown is representative of three independent experiments with similar results. (**f**) Human THP-1 macrophages were infected with RSV (MOI = 1) the presence of DMSO (vehicle control), RIPK3-dependent necroptosis inhibitor GSK’872 (40 µM), Caspase-1 dependent pyroptosis inhibitor ZVAD-FMK (50 µM), or the combination of these inhibitors (GSK ‘872 + ZVAD-FMK). LDH release was measured (at OD of 450 nm) at 16 h post-infection (*n* = 14 technical replicates from two independent experiments). * *p* ≤ 0.05 using a Student’s *t*-test.

IL-1β production as a result of RSV-induced ASC-NLRP3 inflammasome activation in macrophages [25,26] and lung epithelial cells [48] has been well-documented. However, the role of ASC-NLRP3 inflammasome in pyroptotic cell death during RSV infection in macrophages remains undetermined. We investigated the potential role of the ASC-NLRP3 inflammasome in RSV-induced pyroptotic cell death through the caspase-1 inhibitor Ac-YVAD-CHO. Caspase-1 is activated by the multimeric ASC-NLRP3 inflammasome complex [49,50]. Treatment of RSV-infected THP-1 macrophages with two concentrations of Ac-YVAD-CHO, a caspase-1 inhibitor, led to a dose-dependent decrease in LDH release. At the highest concentration, lytic cell death decreased in the inhibited cells by 46% (Figure 4a). It is likely that the remaining 55% lytic cell death occurs via necroptosis since inhibition of the necroptotic pathway by RIPK3 inhibitor led to a 54% loss of lytic cell death (Figure 3a). However, the observed decrease in lytic cell death solely with caspase-1 inhibition does indicate the role of the caspase-1 dependent pyroptosis pathway in promoting lytic cell death in RSV-infected macrophages.

### 3.5. RSV Induces ASC-NLRP3 Inflammasome Dependent Pyroptosis

Given the role of caspase-1 in lytic cell death induction during RSV infection of macrophages (Figure 4a), we further sought to determine the role of individual inflammasome complex components in the induction of pyroptosis. The inflammasome is a multimeric protein complex composed of a “backbone” protein NLRP3 (NOD-, LRR-, and pyrin domain-containing protein 3), an adaptor protein ASC (apoptotic-associated speck-like protein containing a CARD) and pro-caspase 1. Activation of the inflammasome first requires priming by a PAMP (pathogen-associated molecular patterns, e.g., LPS) that activates the corresponding PRR (pattern recognition receptor, e.g., toll-like receptor 4 or TLR4). This “first signal” induces upregulation of genes for pro-IL-1β, NLRP3, and other pro-inflammatory cytokines [49,50]. If a second signal is then encountered (e.g., reactive oxygen species, K+ efflux, etc.), the inflammasome components assemble through CARD-CARD and PYD-PYD domain interactions [49,50]. In the case of the ASC adaptor protein, this CARD-CARD interaction allows cleavage of pro-caspase-1 to generate active caspase-1. While caspase-1 is considered the rate-limiting step in the cleavage of pro IL-1β to active IL-1β, its other role involves the cleavage of Gasdermin-D and induction of pyroptotic cell death. Despite the dual role of caspase-1, production of IL-1β is not intrinsically linked to eventual pyroptotic cell death.

To further characterize the role of inflammasome activation in inducing pyroptotic lytic cell death of RSV-infected macrophages, we used ASC- or NLRP3-deficient THP-1 cells and THP-1 control (wild type null) cells. ASC- and NLRP3- deficient cells are defective in inflammasome activation and we utilized these cells previously to assess the role of the inflammasome during human parainfluenza virus-3 infection [51]. As expected, due to the inability of ASC- and NLRP3- deficient cells to activate the inflammasome, these cells failed to produce IL-1β in the presence of the well-known ASC-NLRP3 inflammasome inducer LPS (first signal) and nigericin (second signal) (Figure 4b). Similarly, infection of these cells with RSV yielded a drastic loss of IL-1β production since NLRP3 and ASC are required for RSV-mediated inflammasome activation (Figure 4c) [25]. To evaluate the role of the NLRP3-ASC inflammasome in RSV induced lytic cell death, we infected ASC- or NLRP3-deficient THP-1 cells and THP-1 control null cells with RSV and measured LDH release. NLRP3-deficient and ASC-deficient cell infection resulted in drastic loss of lytic cell death (Figure 4d), consistent with their central role in the inflammasome and caspase-1 activation. LDH release was inhibited by 72–75% in ASC- and NLRP3-deficent macrophages (Figure 4d). To investigate the infection status of these cells, we infected control null, ASC-deficient, and NLRP3-deficent cells with mKate2-expressing RSV (mKate-2-RSV) [36,37]. We detected similar levels of mKate2 protein in deficient macrophages and control cells (Figure 4e). Therefore, loss of IL-1β production (Figure 4c) and LDH release (Figure 4d) from RSV-infected ASC- and NLRP3-deficient cells were not due to reduced infectivity of these cells compared to the control (null) cells. These results demonstrate the critical role of ASC-NLRP3 inflammasome-dependent caspase-1 activation in promoting pyroptotic cell death in RSV-infected macrophages. It is important to mention that the lack of ASC and NLRP3 led to 72–75% inhibition (Figure 4d) in LDH release compared to the approximately 54% inhibition (Figure 3a) observed with necroptosis inhibitor targeting RIPK3. Thus, it is plausible that the ASC-NLRP3 inflammasome may also partially contribute (by 20–25%) to activation of the necroptosis pathway during RSV infection. Indeed, a role of ASC-NLRP3 inflammasome in inducing RIPK3-MLKL mediated necroptosis has been recently reported [45].

The pan-caspase inhibitor ZVAD-FMK inhibits pro-apoptotic caspase-3 and pro-pyroptotic caspase-1. ZVAD-FMK also blocks caspase-8, an inhibitory caspase in the necroptotic pathway. Therefore, blocking caspase-8 activity by ZVAD-FMK leads to induction of necroptosis through removal of its inhibitory activity on RIPK3 [52,53]. Despite this potential inductive effect, ZVAD-FMK (50 µM) treatment led to a marked decrease in LDH release (Figure 4f). We observed 52% lytic cell death inhibition with ZVAD-FMK treatment. The combined roles of pyroptosis and necroptosis were further investigated through treatment of RSV-infected macrophages with a combination of GSK’872 (40 µM) and ZVAD-FMK (50 µM), addressing both RIPK3-dependent necroptosis and caspase-1 dependent pyroptosis in induction of lytic cell death, respectively (Figure 4f). The treatment of infected macrophages with the combination of these inhibitors was compared to the effect of individual inhibitor treatment alone. Inhibition of RIPK3 via GSK’872 resulted in a 47% reduction in LDH release (Figure 4f) consistent with previous results highlighting the significance of RIPK3 in induction of necroptosis (Figure 3a). Treatment with ZVAD-FMK resulted in a 52% reduction in LDH release (Figure 4f). Treatment of RSV-infected macrophages with the combination of these inhibitors, however, resulted in a 77% reduction in lytic cell death (Figure 4f). Together, our studies have uncovered the RIPK3-MLKL necroptotic pathway and ASC-NLRP3-caspase-1 pyroptotic pathway as key mechanisms that facilitate lytic cell death of macrophages during RSV infection.

### 3.6. Role of ROS in RSV-Induced Lytic Cell Death

**Figure 5:**
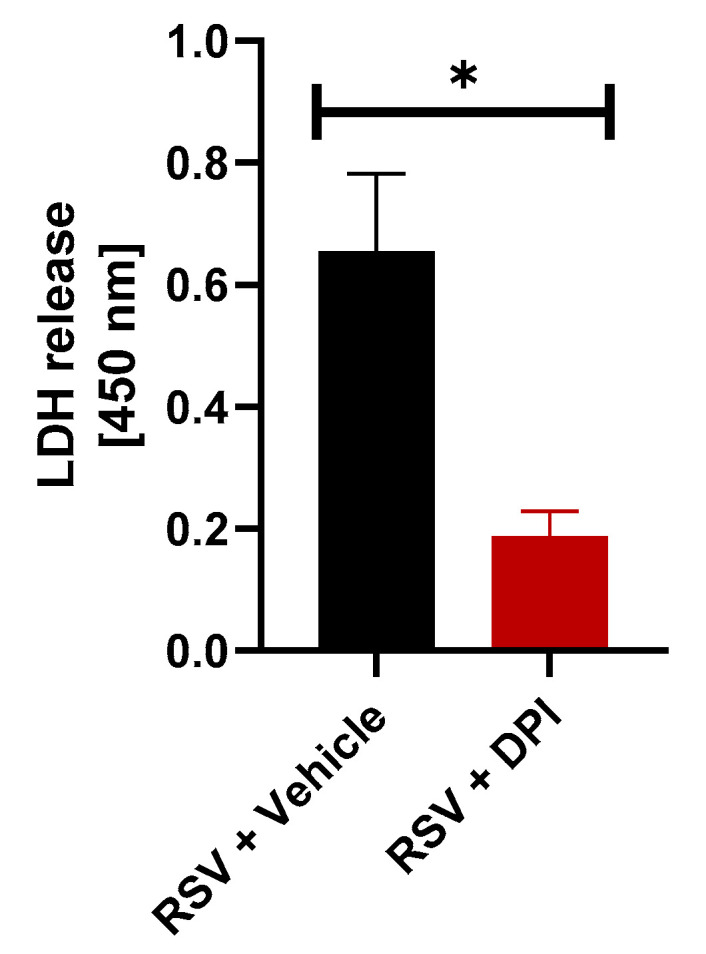
Reactive oxygen species (ROS) regulates lytic cell death of RSV-infected macrophages. Human THP-1 macrophages were infected with RSV (MOI = 1) in the presence of either vehicle (DMSO) or ROS inhibitor diphenyleneiodonium chloride (DPI) (10 µM). LDH release was measured (at OD of 450 nm) at 16 h post-infection (*n* = 16 technical replicates from two independent experiments). * *p* ≤ 0.05 using a Student’s *t*-test.

Reactive oxygen species (ROS) such as hydrogen peroxide and hydroxyl ions are created through the reduction of oxygen during molecular processes. ROS play an important role in both pyroptosis and necroptosis [54,55,56,57,58,59,60]. In pyroptosis, ROS commonly serve as the second signal leading to inflammasome activation, which forms as a precursor to caspase-1 activation, Gasdermin D cleavage, and subsequent membrane rupture. ROS is induced during RSV infection and has been previously shown to trigger NLRP3 inflammasome activation in RSV-infected macrophages [25]. Additionally, recent studies have illustrated that ROS positively regulates both RIPK1-dependent [54,59] and RIPK1-independent [24] necroptosis following activation of RIPK3-MLKL pathway. Thus, it is possible that ROS may also regulate lytic cell death processes during RSV infection in macrophages by modulating both pyroptosis and necroptosis. To determine if inhibition of ROS would dampen lytic cell death, THP-1 cells were treated with diphenyleneiodonium chloride (DPI), a potent ROS inhibitor. Blocking ROS during RSV infection resulted in a 72% decrease in LDH release (Figure 5). This result highlighted the pivotal role of ROS in promoting lytic cell death in RSV-infected macrophages.

## 4. Discussion

RSV is an enveloped, single stranded, non-segmented, and negative-sense RNA-encoding virus in the Pneumoviridae family. RSV is a major cause of inflammatory respiratory disease in at-risk populations including infants, toddlers, the elderly, and immunocompromised people worldwide [1,2,3]. Secondary bacterial infections frequently exacerbate clinical disease through amplified inflammation, accumulation of necrotic epithelial and immune cellular debris, and pulmonary edema resulting in extended hospitalizations and even death. Cellular debris generated due to cell lysis directly contributes toward physical bronchiolar obstruction [15]. In addition, the release of cellular components (e.g., ATP, S100A9 protein, 25-hydroxycholesterol) during cell lysis act as DAMPs to further drive the amplification of inflammation through activation of pro-inflammatory signaling cascades in the surrounding tissue-resident cells [8,16,17,35]. Together, this positive feedback cycle results in plugs of accumulating dead epithelial and immune system cells, their cellular fragments and recruited inflammatory cells within the lumen of airways. Given the lack of a vaccine despite extensive efforts and few effective anti-viral treatments, management of RSV-induced bronchiolitis and pneumonia may rest in treatment of the response rather than the cause.

RNA viruses like influenza A virus induce lytic cell death via both pyroptosis and necroptosis [61,62,63]. However, the exact mechanism of lytic cell death in RSV-infected macrophages was unknown. In this study, we investigated the individual roles of pyroptosis and necroptosis in lytic cell death of macrophages during RSV infection. Neutrophils, the other major immune cell recruited in RSV infection, have recently been shown to undergo necroptosis after infection [27]. This same study showed that RSV induces the production of ROS in neutrophils. Although macrophages are indispensable for the early innate immune inflammatory response during RSV infection, no studies thus far have characterized the lytic cell death pathways or the role of ROS in their induction during RSV infection of macrophages. In the current study, we identified both an ASC-NLRP3 inflammasome-caspase 1 dependent pyroptotic pathway and RIPK3-MLKL necroptotic pathway contributing to lytic cell death of RSV-infected macrophages. These studies suggest an important role of both necroptosis and pyroptosis in contributing to RSV-associated airway disease by amplifying lung inflammation through the generation of cellular debris following lysis of RSV-infected macrophages.

Cell death mechanisms are categorized as either non-lytic and therefore non-inflammatory or lytic and therefore pro-inflammatory, respectively. Apoptosis is the best characterized of the non-lytic cell death processes. Little, if any, inflammation is generated from this form of cell death. In contrast, the mechanisms of both pyroptosis and necroptosis are lytic and therefore pro-inflammatory. However, these mechanisms have distinct differences in their molecular machinery that create potential opportunities for drug target development. Pyroptosis occurs as a sequela of inflammasome mediated downstream activity of caspase-1 on Gasdermin D, the central component of pyroptotic membrane pore formation, leading to osmotic-induced membrane rupture [12,19]. Necroptosis occurs through the activity of the necroptosome, a complex of three key proteins: receptor-interacting serine/threonine-protein kinase 1 (RIPK1), receptor-interacting serine/threonine-protein kinase 3 (RIPK3), and mixed lineage kinase domain like pseudokinase (MLKL) [13,20,21]. Central to necroptosis is the stepwise phosphorylation of each of these components. Autophosphorylation of RIPK1 results in the phosphorylation and activation of RIPK3. Activated RIPK3 in turn recruits and phosphorylates MLKL, which oligomerizes to form the pore-forming compound that migrates to the cell membrane and creates pores like that in pyroptosis. However, necroptosis can also be induced by a RIPK1- independent mechanism, whereby RIPK3 complexes with either TRADD, DAI, or TRIF to self-activate, culminating in MLKL activation and necroptosis [22,23,24]. Additionally, the ASC-NLRP3 inflammasome can also directly activate RIPK3 (independent of RIPK1) leading to MLKL activation and subsequent necroptosis [45]. Current in vivo therapy development efforts have revolved around inhibition of principally necroptosis and its key components RIPK3 and MLKL [64,65,66,67]. Early study highlighted the beneficial effects of RIPK3 and MLKL inhibitors on inflammation in animal models of disease ranging from ischemic injury to autoimmune disorders and neoplasia [68,69]. Further investigation in the use of these types of pharmaceuticals in infectious disease such as RSV-induced pneumonia is warranted given the poor progress in vaccine development.

In the current study, we demonstrated that both pyroptosis and necroptosis are integral pathways in the lytic cell death of macrophages infected with RSV. Using a caspase-1 inhibitor, we highlighted the role of this enzyme in lytic cell death during pyroptosis. We also used NLRP3 and ASC deficient THP-1 cells to demonstrate a key role of the NLRP3-ASC inflammasome in induction of pyroptosis. In the studies involving the necroptosis pathway, we utilized inhibitors of RIPK3 and MLKL and demonstrated that each is significant in the induction of lytic cell death in RSV-infected macrophages. We further investigated the role of ROS, a second signal for inflammasome activation and positive modulator of RIPK3. Our studies revealed a critical role of ROS in promoting lytic cell death pathways in RSV-infected macrophages.

Interestingly, both ASC- and NLRP3-deficient macrophages exhibited 72–75% loss of lytic cell death following RSV infection (Figure 4d). In contrast, loss of lytic cell death with necroptosis inhibitor (i.e., RIPK3 inhibitor) and pyroptosis inhibitor (i.e., caspase-1 inhibitor) was 54% and 46%, respectively (Figure 3a and Figure 4a). Thus, inhibition of lytic cell death with ASC- and NLRP3-deficient cells was relatively higher compared to loss of lytic cell death following inhibition of RIPK3-mediated necroptotic pathway. In light of a recent study showing RIPK3-MLKL necroptotic pathway induction by ASC-NLRP3 inflammasome independent of caspase-1 [45], our result suggests a possibility of ASC-NLRP3 inflammasome not only inducing pyroptosis during RSV infection but partially contributing to necroptosis induction during infection via caspase-1 independent mechanism. It is important to note that blocking pyroptosis by using caspase-1 inhibitor reduced lytic cell death by 46% (Figure 4a), which may infer that 46% inhibition in lytic cell death observed with ASC- and NLRP3-deficent cells could be due to loss of pyroptosis, while the rest 26–29% could be due to loss of necroptosis. Furthermore, it should be taken into consideration that difference in inhibition level could arise due to two different approaches (i.e., using inhibitor treated cells vs. using deficient cells) utilized to investigate pyroptosis vs. necroptotic lytic cell death. Thus, our results suggest that RSV induced lytic cell death in macrophages occurs by both necroptosis and pyroptosis following activation of the RIPK3-MLKL and ASC-NLRP3 inflammasome/caspase 1 pathways, respectively. Furthermore, ASC-NLRP3 inflammasome may also play a role in partially inducing necroptosis via caspase-1 independent mechanism.

Based on our results, we postulate several mechanistic scenarios leading to lytic cell death in RSV-infected macrophages via pyroptosis and necroptosis. RSV activates TLR2 in macrophages and triggers ROS generation in infected macrophages [25]. In fact, both TLR2 and ROS are required for ASC-NLRP3 inflammasome activation in RSV-infected macrophages [25]. Thus, TLR2 activation by RSV and ROS generation during infection will promote formation of ASC-NLRP3 inflammasome complex that activates caspase-1 for Gasdermin-D mediated pyroptosis. RSV also activates TLR4 [70,71] and TLR3 [72] in macrophages. TLR4 and TLR3 activation triggers a RIPK1-independent, TRIF-dependent RIPK3-MLKL necroptotic pathway [22]. Thus, activation of TLR4 and TLR3 by RSV may result in formation of a RIPK1-independent, RIPK3-TRIF complex that activates MLKL for necroptosis induction. Furthermore, drastic reduction in lytic cell death in RSV-infected ASC- and NLRP3-deficent macrophages suggests that the ASC-NLRP3 inflammasome may also play a partial role in inducing necroptosis. This possibility exists since the ASC-NLRP3 inflammasome activated the RIPK3-MLKL necroptotic pathway [45]. This occurred in caspase-1 deficient or caspase-1 inhibited cells [45]. Viruses like influenza A virus and vaccinia virus encode proteins (e.g., NS1 of influenza A, serpins of vaccinia virus) that can inactivate caspase-1 [73]. Thus, RSV may also encode protein(s) that disrupt caspase-1 activity during infection, thus triggering an ASC-NLRP3 inflammasome mediated activation of the necroptotic pathway. In the future we will study whether RSV NS1 protein can also disrupt caspase-1 activity similar to influenza A NS1 protein. Additionally, analogous to its role in pyroptotic pathway, ROS generated during RSV infection may contribute to necroptotic cell death via a RIPK1-independent, TRIF-dependent RIPK3-MLKL necroptotic pathway, as shown previously [24].

In summary, our studies have demonstrated that both pyroptosis and necroptosis pathways are involved in lytic cell death during RSV infection of macrophages and that ROS generated during infection are involved in positively regulating lytic cell death of RSV-infected macrophages.
